# *Sclerotinia sclerotiorum* *SsCut1* Modulates Virulence and Cutinase Activity

**DOI:** 10.3390/jof8050526

**Published:** 2022-05-20

**Authors:** Yingdi Gong, Yanping Fu, Jiatao Xie, Bo Li, Tao Chen, Yang Lin, Weidong Chen, Daohong Jiang, Jiasen Cheng

**Affiliations:** 1 State Key Laboratory of Agricultural Microbiology, Huazhong Agricultural University, Wuhan 430070, China; yingdigong341@163.com (Y.G.); jiataoxie@mail.hzau.edu.cn (J.X.); boli@mail.hzau.edu.cn (B.L.); taochen@mail.hzau.edu.cn (T.C.); daohongjiang@mail.hzau.edu.cn (D.J.); 2 The Provincial Key Lab of Plant Pathology of Hubei Province, College of Plant Science and Technology, Huazhong Agricultural University, Wuhan 430070, China; yanpingfu@mail.hzau.edu.cn (Y.F.); yanglin@mail.hzau.edu.cn (Y.L.); 3 United States Department of Agriculture, Agricultural Research Service, Washington State University, Pullman, WA 99164, USA; w-chen@wsu.edu

**Keywords:** *Sclerotinia sclerotiorum*, cutinase, virulence, defense response

## Abstract

The plant cuticle is one of the protective layers of the external surface of plant tissues. Plants use the cuticle layer to reduce water loss and resist pathogen infection. Fungi release cell wall-degrading enzymes to destroy the epidermis of plants to achieve the purpose of infection. *Sclerotinia sclerotiorum* secretes a large amount of cutinase to disrupt the cuticle layer of plants during the infection process. In order to further understand the role of cutinase in the pathogenic process of *S. sclerotiorum*, the *S. sclerotiorum* cutinsae 1 (*SsCut1*) gene was cloned and analyzed. The protein SsCut1 contains the conserved cutinase domain and a fungal cellulose-binding domain. RT-qPCR results showed that the expression of SsCut1 was significantly upregulated during infection. Split-Marker recombination was utilized for the deletion of the *SsCut1* gene, Δ*SsCut1* mutants showed reduced cutinase activity and virulence, but the deletion of the *SsCut1* gene had no effect on the growth rate, colony morphology, oxalic acid production, infection cushion formation and sclerotial development. Complementation with the wild-type *SsCut1* allele restored the cutinase activity and virulence to the wild-type level. Interestingly, expression of *SsCut1* in plants can trigger defense responses, but it also enhanced plant susceptibility to *SsCut1* gene knock-out mutants. Taken together, our finding demonstrated that the *SsCut1* gene promotes the virulence of *S. sclerotiorum* by enhancing its cutinase activity.

## 1. Introduction

*Sclerotinia sclerotiorum* is a necrotrophic pathogen with a wide range of hosts, causing diseases and resulting in huge economic losses in many important crops such as oilseed rape, soybean, sunflower, and tomato [1]. Studies have shown that the pathogenicity mechanisms of *S. sclerotiorum* are very complicated. Many factors play important roles in the process of *S. sclerotiorum* infection, such as different plant cell wall-degrading enzymes and oxalic acid [2,3,4,5,6]. In addition, secreted proteins also play key roles in the pathogenic process of *S. sclerotiorum*, including SsCaf1, SsSSVP1, SsCP1 and SsITL [7,8,9,10]. At present, however, our understanding of the pathogenesis of *S. sclerotiorum* is still insufficient, and further research on its pathogenesis contributes to the development of new control strategies for Sclerotinia diseases [11,12,13].

The plant cuticle covers the leaf epidermis of higher plants and is the main barrier that isolates plants from direct contact with the atmosphere. The plant cuticle is mainly composed of cutin and wax, which is a hydrophobic property [14,15,16,17,18,19]. For different plant species, the composition of cutin and wax is quite different [20], but the ability to synthesize hydrophobic surface layers is evolutionarily conserved [16,18,21]. The plant cuticle is the first line of defense against pathogens, because the cuticle covers the outermost layer of the plant epidermis. The plant cuticle can resist pathogen invasion and endow the corresponding microbial environment [19,22,23,24,25,26,27,28,29]. Interestingly, many studies have shown that the cuticle is not only a physical barrier of protection, but also participates in plant growth and development, immunity, and signal transduction [30,31,32,33,34].

The plant cuticle is an important component for plant–fungi interactions, so alterations to its structure and permeability might change immunity to pathogenic fungi [17,35]. Most plant pathogens produce cutinase to destroy the plant cuticle to facilitate their infection [36,37,38,39,40]. Cutinase (EC 3.1.1.74) is a serine esterase belonging to the α/β hydrolase superfamily, capable of breaking down cutin polyesters. Most cutinases contain a conserved GYSQG domain and the similar DxVCxG [ST]-[LIVMF] (3)-x (3) H motif. They have the classic Ser–His–Asp catalytic triad and can hydrolyze a variety of substrates, including low-molecular-weight soluble esters as well as short- and long-chain triacylglycerols. Cutinase can also catalyze esterification and transesterification [37,41].

Although cutinases share similarities in conserved domains and amino acid sequences, cutinases from fungi of different lifestyles vary in properties such as molecular weight, optimum temperature and pH optimum for action, substrate specificity and thermostability [42]. Cutinase is able to degrade cutin polymers, thereby destroying the physical properties of the plant epidermis [29]. So cutinase plays important roles during the infection process of many plant pathogenic fungi. For example, disruption of cutinase gene *Pbc1* can lead to the loss of virulence in *Pyrenopeziza brassicae* [43]. In the corn leaf pathogen *Curvularia lunata*, deletion of *Clcut7* also reduced the virulence and cutin-decomposing ability [44]. The virulence of the cutinase gene *CglCUT1* knock-out strain was also decreased in *Colletotrichum gloeosporioides* [45]. However, in some pathogens such as *Nectria haematococca*, *Botrytis cinerea*, *Fusarium graminearum* and *Ustilaginoidea virens*, the knock-out of specific cutinase genes showed no effect on the virulence [46,47,48,49], possibly due to redundant function of multiple cutinase genes. An obvious example is that the knock-out mutants of either *AaCut3* or *AaCut7* have a normal virulence phenotype in *Alternaria alternata*, while the virulence of the double-mutant strains of *AaCut3* and *AaCut7* are significantly reduced [50]. On the other hand, the enhancement of cutinase activity can increase the virulence of pathogenic fungi. The overexpression strain of *MfCUT1* in *Monilinia fructicola* produced larger lesions on *Prunus* flower petals than the wild-type strain [51]. Interestingly, some studies have shown that cutinase can also activate plant immune response during infection. For instance, purified *Verticillium dahliae* protein VdCUT11 can induce cell death in tobacco, cotton and tomato plants and trigger a defense response [52]. The cutinase RcCUT1 of *Rhizoctonia cerealis* can also induce necrosis of plant cells, H_2_O_2_ accumulation and expression of defense-related genes [53]. These results indicate that the role of cutinase in the infection of pathogenic fungi is complicated, and it also participates in the interaction between host and pathogens.

*S. sclerotiorum* secretes a large number of hydrolytic enzymes when infecting plants, including cell wall-degrading enzymes, cutinase and proteases. It was predicted that there are eight genes encoding cutinase proteins in the *S. sclerotiorum* genome [54]. One of the cutinase genes, *SsCUT*, is highly expressed in the early stage of infection. Expression of SsCut in *Nicotiana benthamiana* can induce the death of host cells and activate the plant immune response [55,56]. However, the effect of *SsCut* on the phenotype of *S. sclerotiorum* was not involved in previous studies. Additionally, the functions of other cutinase genes have not been studied in *S. sclerotiorum*. In this study, the expression level, secretory activity and subcellular localization of SsCut1 were clarified. Most importantly, the *SsCut1* gene was knocked out, and the cutinase activity and the virulence of the *SsCut1* knock-out mutants decreased significantly, while the cutinase activity and virulence of the complementary strain all recovered to the level of wild-type strain. In addition, expression of *SsCut1* in *N. benthamiana* plant can complement the lost virulence of the *SsCut1* knock-out mutant. Our results suggest that *SsCut1* plays important roles in the pathogenesis of *S. sclerotiorum*.

## 2. Materials and Methods

### 2.1. Strains and Culture Conditions

The *S. sclerotiorum* (Lib.) de Bary wild-type strain 1980 (ATCC 18683) was obtained from diseased bean plants in Scottsbluff, NE [57]. In this study. Fungal strains were cultured on potato-dextrose agar (PDA) plates at 20 °C. The *SsCut1* gene knock-out mutants and complement mutants were cultured on PDA plates amended with hygromycin B or G418 at 100 µg/mL (Sigma-Aldrich, St. Louis, MO, USA).

*Escherichia coli* DH5α and Top10 (Transgen Biotech, Wuhan, China) for plasmid proliferation was cultured in Luria–Bertani (LB: 1% Tryptone, 0.5% Yeast extract, 0.5% NaCl and 1% agar, pH = 7) medium with appropriate antibiotics (Kanamycin or Ampicillin). The LB medium with appropriate antibiotics (Kanamycin and Rifampicin) was used to culture *Agrobacterium tumefaciens* EHA-105 and GV3101, which was used in fungal transformation and protein expression.

### 2.2. Bioinformatics Analysis

Sequences of SsCut1 were retrieved from the NCBI GenBank database. The cutinase signal peptide sequence was predicted by using SIGNAIP 5.0 tools (http://www.cbs.dtu.dk/services/SignalP/) (accessed on 17 May 2022, DTU, Copenhagen, Denmark). The structural domains of cutinase were predicted by NCBI Conserved Domain Search Tools (https://www.ncbi.nlm.nih.gov/Structure/cdd/wrpsb.cgi) (accessed on 17 May 2022, Bethesda, MA, USA). The cutinase promoter was predicted by using Promoter 2.0 Prediction Server (http://www.cbs.dtu.dk/services/Promoter/) (accessed on 17 May 2022, DTU Bioinformatics, Copenhagen, Denmark). The cutinase transmembrane domains were predicted by using TMHMM Server, v. 2.0 (http://www.cbs.dtu.dk/services/TMHMM/) (accessed on 17 May 2022, DTU Bioinformatics, Copenhagen, Denmark). Other cutinase sequences were also retrieved from the NCBI GenBank database. Amino acid sequence alignments were generated via the ES Pript 3.0 Tools (https://espript.ibcp.fr/ESPript/cgi-bin/ESPript.cgi) (accessed on 17 May 2022). The phylogenetic tree was constructed with MEGA 6.0 [58] using the maximum-likelihood method.

### 2.3. DNA/RNA Manipulation and RT-qPCR

Genomic DNA was isolated from mycelium by using the CTAB method [59]. Total RNA of fungi and plants were isolated with RNAiso Plus regent (Takara, Dalian, China) according to the manufacturer’s protocols. To detect the expression pattern of cutinase gene in infected tissues, the hyphae of *S. sclerotiorum* wild-type strain were inoculated on *Arabidopsis thaliana* plant leaves. The hyphae were collected at 0, 1, 3, 6, 9, 12 and 24 h after inoculation. After RNA extraction, the first-strand cDNA was synthesized by the Easy Script One-Step gDNA Removal and cDNA Synthesis SuperMix (TransGen Biotech, Beijing, China). Additionally, the gene expression was detected by qPCR analysis [60]. The *β-tubulin* gene in *S. sclerotiorum*, the *UBQ5* gene in *A. thaliana*, and the elongation factor *EF1α* gene in *N. benthamiana* were used as internal controls, respectively. The real-time RT-qPCR analysis was repeated at least three times, with three biological replicates for each repeat.

### 2.4. The Yeast Secretion Trap Screen Assay

The secretion activity of SsCut1 was analyzed by using the yeast secretion trap assay [61,62]. In this study, the predicted signal peptide fragment of the *SsCut1* gene was fused to the N-terminal of secretion-defective invertase gene (suc2) in the vector pSUC2 and Then transformed into the yeast competent strain YTK12. The candidate yeast transformants were cultured on YPDA medium, and selected on CMD-W (Takara, Dalian, China) medium and YPRAA (2% raffinose) medium (Macklin, Shanghai, China). TTC (2, 3, 5-Triphenyte-trazoliumchloride) was used to detect the activity of the secreted sucrase of the candidate yeast transformants. The candidate yeast transformed strain was cultured on sucrose medium, then collected by centrifugation, added with a final concentration of 0.1% TTC reagent, incubated at 35 °C for 35 min, and placed at room temperature for 5 min to observe the color change in the test tube. A positive reaction changes from colorless to dark red. The SPs of Avr1b and YTK12-pUSC2 were used as the positive and negative controls, respectively.

### 2.5. Gene Deletion and Complementation

The cutinase gene *SsCut1* was disrupted by using the split marker system [63]. The strategy for the deletion of *SsCut1* is illustrated in Supporting Information Figure S2A. To generate *SsCut1*-deletion mutants, the 5′ and 3′ flanking fragments of the ORF of *SsCut1* were amplified from *S. sclerotiorum* wild-type strain 1980 genomic DNA, then cloned into the pMD19-T plasmid using the T/A clone ligation kit (Takara, Shiga, Japan). Two flanking sequences with a truncated hygromycin-resistant gene sequence were named 5′-HY and YG-3′, respectively. The 5′-HY and YG-3′ fragments were amplified and purified. The purified fragments of 5′-HY and YG-3′ were mixed in equal molarity, and used for protoplast transformation [64]. ∆*SsCut1* mutants were selected on plates containing hygromycin and confirmed by PCR with genomic DNA as template. Homokaryotic ∆*SsCut1* mutants were obtained by single ascospore isolation. The homokaryotic state of the ∆*SsCut1* mutants was confirmed by Southern blot. The genomic DNA was extracted from mycelium and digested with SpeI. Southern blot analysis with SsCut1 as a probe was detected by using Amersham AlkPhos Direct Labelling and Detection Systems (GE Healthcare, Piscataway, NJ, USA).

For the complementation of *SsCut1*, the binary vector pCENTS-SsCut1 was constructed. The complementary fragment was amplified from the *S. sclerotiorum* genomic DNA, including the open reading frame of *SsCut1* with an approximately 1200 bp native promoter region and a 200 bp terminator region. The pCENTS was cut by XhoI and SpeI to remove the EF-1α promoter and then ligated with the complementary fragment. The complementary vector was transformed into *Agrobacterium* strain EHA105. *Agrobacterium tumefaciens*-mediated transformation (ATMT) of *S. sclerotiorum* was performed as described [65]. Cultivate the *Agrobacterium* broth and the mycelium blocks of cutinase knock-out mutants in the dark condition for three days. Then, cover with the PDA medium containing hygromycin B and G418 at 100 µg/mL resistance. The DNA of candidate complementary transformants were extracted by using the CTAB method for validation.

### 2.6. Plant Growth and Pathogenicity Assays

The *A. thaliana* and *N. benthamiana* were grown at 20 °C in growth chambers or glasshouse under long day (16 h: 8 h, light: dark) conditions. Pathogenicity assays of *S. sclerotiorum* were tested with mycelia plugs (diameter = 4.0 mm) from the margins of actively growing colonies on PDA medium. Inoculated tissues were incubated at 21 °C for 48 h before image acquisition and measurement of lesion area. The treatment was replicated 10 times and the experiments were repeated three times.

### 2.7. Generation of Plant Expression Plasmids

In order to express *SsCut1* in *N. benthamiana*, a plasmid with the backbone of pCNF3 and pCNG were used [9]. The recombinant plasmid pCNF3-SsCut1 and pCNG-SsCut1 were transformed into *Agrobacterium* strains GV3101 for expression. The protein of *N. benthamiana* were extracted for Western blot at 48 h post-infiltration. The leaf specimens of *N. benthamiana* were prepared to observe the protein subcellular localization by using the confocal microscope (Olympics).

### 2.8. Cutinase Activity Assay

The ELISA Reagent test kit (Sinobestbio, Shanghai, China) was used to determine the cutinase activity of fungal hypha samples. The HRP-labeled detection antibody was added to the coated microwells pre-coated with cutinase antibody, incubated and washed thoroughly. The color is developed with the substrate TMB. TMB is converted into a blue color under the catalysis of peroxidase, and converted into the final yellow under the action of acid. The intensity of the color is positively correlated with the cutinase activity in the sample. The absorbance (OD value) at a wavelength of 450 nm was measured with a microplate reader to calculate the cutinase activity in the sample.

## 3. Results

### 3.1. SsCut1 Contains Cutinase Domain and Fungal Cellulose-Binding Domain

**Figure 1:**
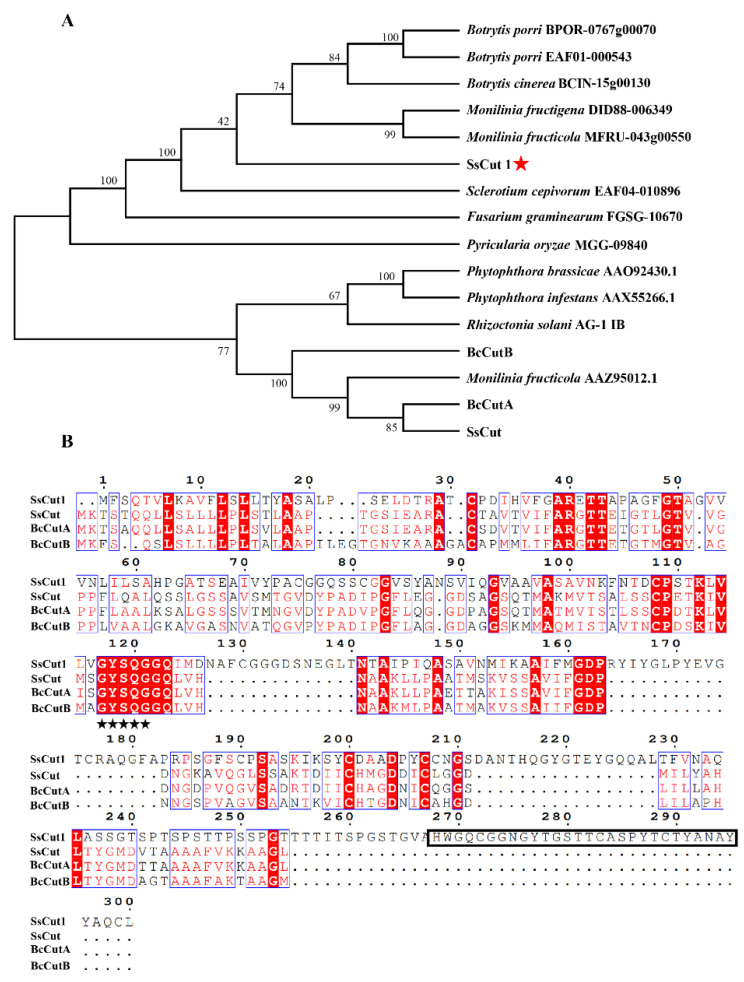
Analysis of the cutinase protein SsCut1. (**A**) The evolutionary relationship of SsCut1 and its homologs from other fungi determined with the maximum-likelihood algorithm. Branch lengths are proportional to the average probability of change for characters on that branch. The phylogeny was constructed with Mega 6.0 using the neighbor-joining method (parameters: 1000 bootstraps). (**B**) The amino acid sequence alignment of SsCut1, SsCut, BcCutA and BcCutB. The black stars indicate the conserved GYSQG catalytic site. The rectangle box indicates the conserved CBM1 domain.

**Figure 2:**
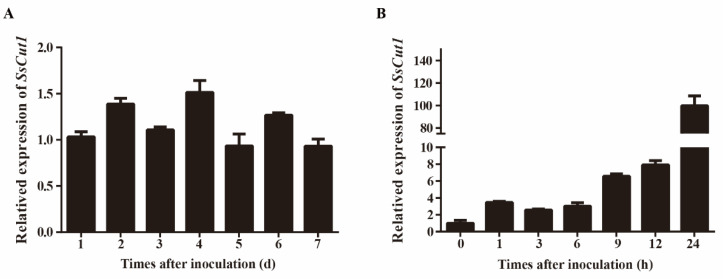
Expression patterns of *SsCut1* of *S. sclerotiorum* at different stages of *S. sclerotiorum*. (**A**) Expression patterns of the *SsCut1* in culture on PDA medium at 20 °C for 1–7 d (days). (**B**) Expression patterns of the *SsCut1* during the infection of *A. thaliana* at 20 °C for 0–24 h (hours). The *S. sclerotiorum β-tubulin* gene was used as an internal control to normalize the data. Error bars represent the standard error (*n* = 3).

Sequence analysis showed that there are eight genes encoding cutinase proteins in the genome of *S. sclerotiorum* (Supplementary Figure S1). *SS1G_08104* is one of the cutinase-encoding genes, which was named *SsCut1* in this study. *SsCut1* is 1077 bp in length and contains three exons and two introns. Its open reading frame encodes a 300 aa protein with a predicted N-terminal SP. SsCut1 possesses fourteen cysteine residues. Phylogenetic analysis showed that the homologous protein of SsCut1 was widely found in *B. cinerea* and other pathogenic fungi, but it did not cluster with the reported cutinase SsCut, BcCutA and BcCutB (Figure 1A). Protein structure analysis showed that the SsCut1 had conserved GYSQG catalytic site; however, it differed from SsCut, BcCutA and BcCutB in in certain features, consistent with the results of cluster analysis. SsCut1 contains a fungal cellulose-binding (CBM1) domain which was absent in SsCut, BcCutA and BcCutB (Figure 1B). The expression pattern of *SsCut1* was detected by RT-qPCR. The results revealed that the expression of SsCut1 was relatively stable during the growth and development stage of S. sclerotiorum (Figure 2A). However, during the infection stage, the transcript level of SsCut1 was upregulated and increased by more than 100 fold at 24 h post-inoculation (hpi) on *A. thaliana* (Figure 2B), suggesting that *SsCut1* may be involved in infection by *S. sclerotiorum*.

### 3.2. SsCut1 Is a Secreted Protein and Localized in the Plant Cell Wall

**Figure 3:**
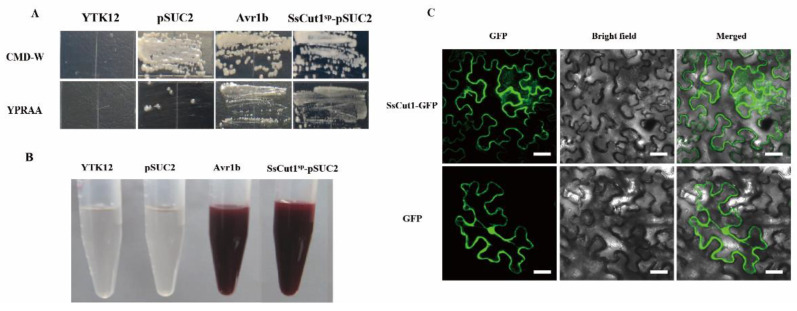
SsCut1 has a functional signal peptide and is localized in the plant cell wall. (**A**) Validation of the secretion function of the SsCut1 signal peptide by the yeast secretion trap screen assay. Signal peptide of SsCut1 was fused in frame to the yeast invertase sequence in pSUC2 vector and expressed in YTK12 strains. The functional signal peptide of Avr1b was used as a positive control, while the YTK12 and pSUC2 empty plasmid was used as a negative control. (**B**) The invertase activity in TTC medium. TTC encounters raffinose breakdown products to produce triphenylformazan, which shows a red reaction to confirm that a functional signal peptide can cause sucrose invertase to be secreted. (**C**) Subcellular localization of SsCut1 in *N. benthamiana* epidermal cells. SsCut1-GFP localized in the plant cell wall. The fluorescence of GFP was monitored at 2 d post-agroinfiltration using confocal laser scanning microscopy. Bar = 20 µm.

To investigate whether SsCut1 is normally secreted by *S. sclerotiorum*, the function of SsCut1 signal peptide was verified by the yeast secretion trap system [61,62]. The Avr1b was used as positive control and the YTK12 empty strain and YTK12 with pSUC2 vector were used as negative controls. The invertase mutant yeast strain YTK12 of pSUC2-Avr1b and pSUC2-SsCut1sp could regularly grow on CMD-W and YPRAA media (Figure 3A). When treating with 2, 3, 5 triphenyltetrazolium chloride (TTC), the mutant strains of pSUC2-Avr1b and pSUC2-SsCut1sp generated the insoluble red-colored triphenylformazan, but the yeast strains YTK12 and pSUC2 remained colorless after treatment (Figure 3B). These results indicated that the signal peptide of SsCut1 was functional in the yeast secretion trap system, and SsCut1 might be a secreted protein.

SsCut1 contains a fungal cellulose-binding domain of CBM1 superfamily at the 268–296 amino acid positions (Figure 1B). This domain has high specific affinity to cellulosics [66]. We speculated that SsCut1 could localize in the plant cell wall. In order to clarify the localization of SsCut1 in plant cells, we performed a protein cellular localization assay in *N. benthamiana*. Fluorescence observation results showed that SsCut1 can be localized in the cell wall when expressed in plant cells (Figure 3C).

### 3.3. SsCut1 Is Important for Virulence of S. sclerotiorum

The biological function of *SsCut1* in *S. sclerotiorum* was investigated by generating *SsCut1*-deletion mutants, using a homologous recombination strategy (Supplementary Figure S2A). Homokaryotic Δ*SsCut1* mutants were obtained by single ascospore isolation. Three *SsCut1*-deletion mutants (Δ*SsCut1-1*, Δ*SsCut1-3* and Δ*SsCut1-7*) were obtained, and confirmed by PCR and Southern blot analysis. The results showed that the targeted gene was replaced by a single copy marker gene in mutants Δ*SsCut1-1* and Δ*SsCut1-3* (Supplementary Figure S2B,C). Two complementary transformants, Δ*SsCut1-3-C8* and Δ*ssCut1-3-C12*, were generated by introducing wild-type allele to Δ*SsCut1-3* mutants using the ATMT method (Supplementary Figure S2D).

**Figure 4:**
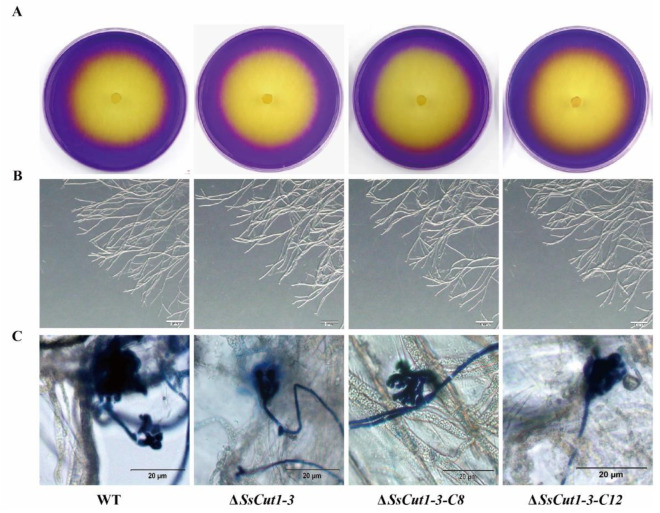
The deletion of *SsCut1* has no significant effect on oxalate production, hypha morphology and infection cushions formation. (**A**) Qualitative determination of acid produced by the wild-type strain and *SsCut1* transformants on PDA medium containing 0.005% (*w*/*v*) bromophenol blue dye as a pH indicator. The presence of yellow indicates that acid was produced. Photographs were taken at 36 hpi. (**B**) In vitro hyphal development of the wild-type strain and *SsCut1* transformants. All strains were cultured on PDA medium for 36 hpi. Hyphal tips were observed under a dissecting microscope. Bars = 500 µm. (**C**) Infection cushions formation of wild-type strain and *SsCut1* transformants. Microscopic observation of infection cushions of wild-type strain and *SsCut1* transformants on onion epidermal cell layer after staining with trypan blue. Photographs were taken at 14 hpi. Bar = 20 µm.

**Figure 5:**
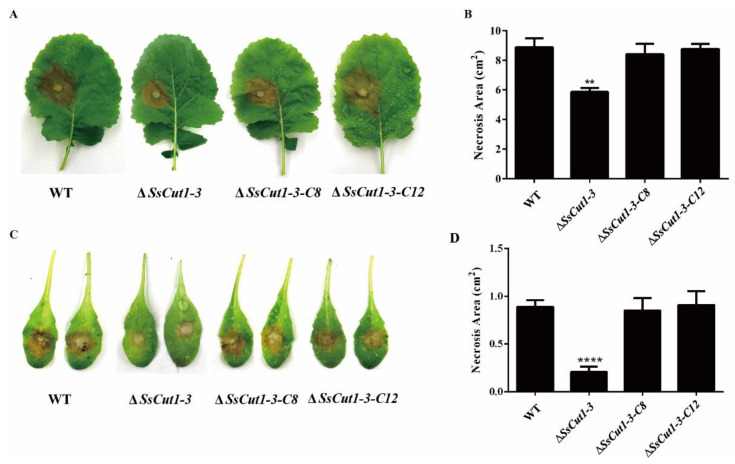
*SsCut1* knock-out mutants showing reduced virulence on the detached leaves of oilseed rape and *Arabidopsis* leaves. (**A**) Lesions formation on oilseed rape leaves inoculated with wild-type strain (1980) and *SsCut1* transformants, the photographs were taken at 48 h post-inoculation (hpi). (**B**) Statistical results of lesion area on oilseed rape leaves. (**C**) Lesions formation on *A. thaliana* leaves inoculated with wild-type strain and *SsCut1* transformants, the photographs were taken at 36 hpi. (**D**) Statistical results of lesion area on *A. thaliana* leaves. Bars indicate ± SE (*n* = 4). Statistical significance is indicated in the graph (one-way ANOVA): ** *p* < 0.01, **** *p* < 0.0001. The experiments were performed three times with similar results.

Deletion of *Sscut1* had no effect on the saprophytic growth and life cycle of *S. sclerotiorum*. Δ*SsCut1* mutants showed no significant differences from the wild-type strain in colony morphology, oxalic acid production, infection cushion formation, sclerotial development and growth rate (Figure 4 and Figure S3). To determine the function of the *SsCut1* in the virulence of *S. sclerotiorum*, the wild-type strain (1980), *SsCut1* knock-out mutants, and the complementary transformants were inoculated on the leaves of oilseed rape and *A. thaliana* plants. Compared with the wild-type strain, both knock-out mutants caused smaller lesions on the oilseed rape and *A. thaliana* leaves. Complementation of *SsCut1* in the knock-out strain restored virulence to the level of the wild-type strain (Figure 5 and Figure S4). These results indicated that *SsCut1* is not required for completion of life cycles, but required for full virulence of *S. sclerotiorum*.

### 3.4. Deletion of SsCut1 Affects the Cutinase Activity of S. sclerotiorum

**Figure 6:**
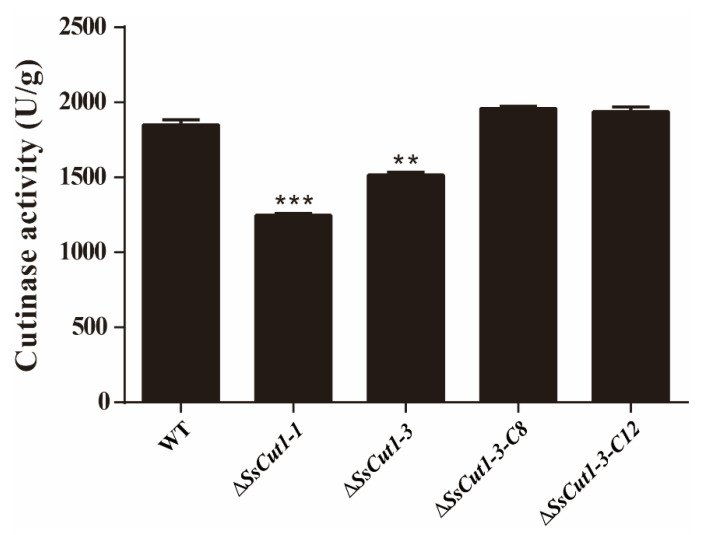
Cutinase activity of wild-type strain and *SsCut1* transformants. All strains were cultured on PDA medium. The hyphal of wild-type strain and *SsCut1* transformants was collected at 36 hpi. Cutinase activity levels were examined with the enzyme-linked immunosorbent assay (ELISA) method. Bars indicate ± SE. Statistical significance is indicated in the graph (one-way ANOVA): ** *p* < 0.01, *** *p* < 0.001.

To investigate if *SsCut1* is associated with the cutinase activity in *S. sclerotiorum*, wild-type strain and different mutants of *SsCut1* were cultured on the PDA medium for 36 h. Then, the hyphae were collected for determination of cutinase activity. Results showed that the cutinase activity of *SsCut1* knock-out transformants were significantly decreased compared with wild-type strain, while the cutinase activity of *SsCut1* complementary transformants were recovered to the level of wild-type strain (Figure 6), indicating *SsCut1* does encode a cutinase in *S. sclerotiorum*. The decrease in virulence of *SsCut1* knock-out transformants should be related to the decrease in cutinase activity.

### 3.5. The Expression of SsCut1 in Plants Triggers Defense Responses

**Figure 7:**
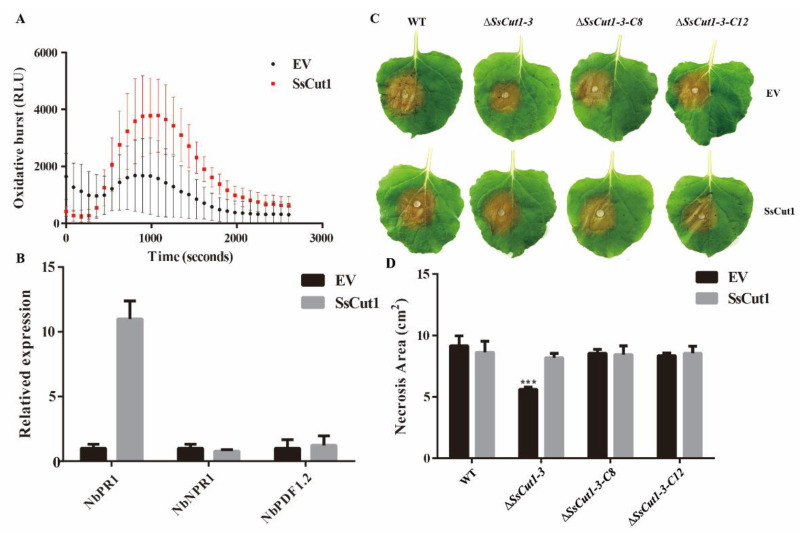
SsCut1 triggers plant defense responses and plays a role in the Sclerotinia–plant interaction. (**A**) SsCut1 promote flg22-triggered reactive oxygen species burst. The *N. benthamiana* with SsCut1 and empty vector were treated with 100 µg/mL flg22. Bars indicate ± SE. Error bars represent the SE from ten biological replicates. (**B**) Induction of defense response genes by SsCut1. SsCut1 induces *NbPR1* expression in *N. benthamiana.* Relative transcript accumulation of *NbPR1*, *NbNPR1*, *NbPDF1.2* genes determined by RT-qPCR. The transcript level of *NbEF1α* in *N. benthamiana* was used to normalize the expression levels in different samples. Error bars represent the SE from three replicates. (**C**) Expression of SsCut1 in *N. benthamiana* increases plant susceptibility to the *SsCut1* knock-out mutant Δ*SsCut1-3*. Leaves of *N. benthamiana* were agroinfiltrated with *Agrobacterium tumefaciens* containing empty vector or pCNF-SsCut1. The wild-type strain and *SsCut1* transformants were inoculated 48 h after agroinfiltration. Photographs were taken at 48 hpi. (**D**) Statistical results of lesion area on *N. benthamiana* leaves. In this experiment, four independent replicates were performed. Bars indicate ± SE. Statistical significance is indicated in the graph (one-way ANOVA): *** *p* < 0.001.

To evaluate whether SsCut1 participates in Sclerotinia–plant interaction, transient expression of *SsCut1* was carried out in *N. benthamiana*. ROS burst was observed when we transiently expressed *SsCut1-flag* in *N. benthamiana* compared with that expressing mock vector (Figure 7A). Furthermore, the expression levels of *NbPR1* were also significantly upregulated when *SsCut1-flag* was expressed (Figure 7B). These results suggested that the expression of *SsCut1* in *N. benthamiana* can trigger plant defense responses. Interestingly, the lesion size of Δ*SsCut1-3* mutant on *SsCut1*-expressing plants was similar to that of the wild-type strain level (Figure 7C, D), suggesting that SsCut1 expressed in plants can complement the virulence defect of the SsCut1-deletion mutant. It is another line of evidence that *SsCut1* is crucial to the virulence of *S. sclerotiorum* and plays an important role in the Sclerotinia–plant interaction.

## 4. Discussion

Cutinase is important for the virulence of many fungal pathogens. The *S. sclerotiorum* genome contains eight cutinase genes [54], but only *SsCut* has been studied [55,56]. However, SsCut was only expressed in *N. benthamiana* to observe the plant defense response, but the effect of SsCut on *S. sclerotiorum* phenotype has not been studied. *SsCut1* encodes a cutinase protein in *S. sclerotiorum* and shows increased expression during the early stage of *S. sclerotiorum* infection (Figure 2), indicated that *SsCut1* plays an important role in the early stage of infection. Therefore, in this study, we explored the effects of *SsCut1* on virulence and cutinase activity of *S. sclerotiorum*.

Most cutinase protein sequences contain a highly conserved GYSQG motif, which is crucial for maintaining the serine activity and function of cutinase [67]. In *S. sclerotiorum*, all the eight cutinase genes contain the conserved GYSQG domain, but the intron and exon structures of the eight cutinase genes are different (Supplementary Figure S1). It is suggested that the cutinase gene in *S. sclerotiorum* genome not only keeps the core GYSQG structure, but also increases the universality and adaptability of cutinase, which is beneficial to the expansion and stability of the host range of pathogenic fungus. The amino acid sequence alignment also shows that although the protein sequences of SsCut1, SsCut, BcCutA and BcCutB are highly conserved in key domains, there are some regions with unknown functions at the C-terminal of SsCut1 (Figure 1), and it is also unknown whether these regions endow new functions to SsCut1.

Plant epidermis layers, mainly composed of cutin, wax, and cellulose, are the first barrier for fungi to infect plants [68,69]. Plant pathogenic fungi, especially necrotrophic fungi, can destroy the integrity of plant cell walls through combined actions of a large number of cell wall-degrading enzymes to facilitate invasion. During this process, cutinase plays key roles and is usually released at the early stage of infection to degrade the plant cuticle, which is the first barrier of plant [70]. Therefore, knocking out cutinase or destroying its activity often has a great influence on the virulence of plant pathogenic fungi. For example, deletion of genes encoding cutinase reduced virulence in *Colletotrichum truncatum*, *C. gloeosporioides*, *Botryosphaeria dothidea* and *Arthrinium phaeospermum* [40,45,71,72]. Using enzyme inhibitors to inhibit cutinase activity can also prevent *Fusarium solani pisi* from infecting plants [73]. We also found that *SsCut1* knock-out transformants have no difference in growth rate, colony morphology, infection cushions formation, sclerotia formation, and oxalic acid production compared with wild-type strain (Figure 4 and Figure S3). However, the virulence and cutinase activity of the *SsCut1* knock-out transformants were significantly reduced, while there was no obvious difference between complementary transformants and wild-type strain (Figure 5 and Figure 6). Considering the high-level expression of *SsCut1* in the *S. sclerotiorum* infection process, our results suggest that the *SsCut1* has cutinase activity and is necessary for full virulence of *S. sclerotiorum*. There are reports that knocking out genes encoding cutinase has no obvious effect on the virulence of some plant pathogenic fungi [46,47,48,49], due to redundancy of gene functions for existence of multiple genes encoding cutinase in the genome. There are also eight cutinase genes in *S. sclerotiorum* [54], which is why the virulence of *S. sclerotiorum* only decreased after *SsCut1* knock-out, but not completely lost.

During the interaction between plants and pathogenic fungi, cutinase can not only degrade the cuticle of plants, but also be recognized by plants as a PAMP (Pathogen-associated molecular patterns), thus activating the immune response [40,52,53,56,74]. For example, overexpression of the cutinase gene *CUTE* (cutinase-expressing) in *Arabidopsis* can improve the resistance of plants to *B. cinerea* [35]. Overexpression of a cutinase gene from *B. dothidea* (*Bdo_10846*) in *N. benthamiana* induced ROS burst and callose deposition, and also enhanced *N. benthamiana* resistance to *B. cinerea* [40]. The SsCut protein of *S. sclerotiorum* can also cause cell death in soybean and rapeseed, and induce resistance to *S. sclerotiorum* and *B. cinerea* in these plants [56]. In this study, we found that the expression of *SsCut1* in *N. benthamiana* induced ROS burst, and upregulated expression of host pathogenesis-related genes (Figure 7A,B). However, when the wild-type strain 1980 was inoculated, the disease lesions on the plants expressing SsCut1 did not increase compared with the empty vector-expression plants, indicating that the resistance of these plants to *S. sclerotiorum* did not change significantly (Figure 7C,D). This may be due to the strong virulence of *S. sclerotiorum* or the existence of multiple cutinase in the genome of *S. sclerotiorum*. SsCut1 and other cutinase in *S. sclerotiorum* may also be recognized by plants during infection, thus inducing plant immune response. For example, the expression of SsCut also can trigger plant immune response [56], so the immune response induced by SsCut1 expression in plants may not be enough to affect the virulence of *S. sclerotiorum*. Interestingly, the virulence of the *SsCut1* knock-out mutant on *SsCut1*-expressing plants recovered to the level of wild-type strain 1980, indicating that *SsCut1* is important to the virulence of *S. sclerotiorum* and plays an important role in plants during infection.

## 5. Conclusions

In summary, our study revealed that *SsCut1* is important for the cutinase activity and full virulence of *S. sclerotiorum*, and plays important roles in triggering plant defense responses. Here, we identified a conserved cutinase gene, *SsCut1* from *S. sclerotiorum*. *SsCut1* was found to be expressed highly in the early stage of *S. sclerotiorum* infection. SsCut1 had secretory activity and was mainly located in the plant cell wall. Knocking out *SsCut1* reduced the virulence and cutinase activity of *S. sclerotiorum*, but had no effect on the growth rate, colony morphology, oxalic acid production, infection cushion formation and sclerotial development. Expression of *SsCut1* in plants can trigger defense responses, such as induced ROS burst and upregulate the expression of pathogenesis-related genes. However, it also enhances plant susceptibility to *SsCut1* gene knock-out mutants. These findings improve our understanding of the roles of cutinase gene during the interaction of *S. sclerotiorum* with its host plants, and also offer clues to further reveal the mechanisms of these genes in infection.

## Data Availability

The data presented in this study are available on request from the corresponding author.

## Supplementary Materials

The following are available online at https://www.mdpi.com/article/10.3390/jof8050526/s1, Figure S1: Exon and intron structure display of cutinase family genes of *S. sclerotiorum*. Exons and introns are indicated by red boxes and lines, respectively. The names of the *S. sclerotiorum* cutinase genes and intron-exon structures are indicated at the left and right sides, respectively. The number in the figures indicates the length of each intron and exon. Figure S2: Analysis of *SsCut1* mutants. (A) Schematic diagram of the *SsCut1* gene knock-out strategy. The different colored parts show the *HptII* cassette (white color parts), *SsCut1* (black box parts) and flanked sequences (grey box parts) (B) PCR validation of the *SsCut1* knock-out strains. Partial *HptII* fragment (lanes 3, 7, 11, 15); the upstream of *SsCut1* overlapped Ptrp (lanes 1, 5, 9, 13); the downstream of *SsCut1* overlapped trpC (lanes 2, 6, 10, 14); the full-length *SsCut1* (lanes 4, 8, 12, 16). Lane M, DL5000 marker (Takara, Dalian, China). (C) Southern blot analysis of the SpeI-digested genomic DNAs from wild-type strain, Δ*SsCut1-1*, Δ*SsCut1-3* and Δ*SsCut1-7* strains. Probe was labelled with alkaline phosphatase. Lane M: DNA marker λ-HindIII DNA Ladder. (Takara, Dalian, China) (D) PCR validation of the *SsCut1* complementary strains. The full-length *SsCut1* (lanes 1, 4, 7, 10); partial *HptII* fragment (lanes 2, 5, 8, 11); partial G418 fragment (lanes 3, 6, 9, 12). Lane M, DL2000 marker (Takara, Dalian, China). Figure S3: Colony morphology of WT, Δ*SsCut1-3*, Δ*SsCut1-3-C8* and Δ *SsCut1-3-C12* strains. (A) Growth of wild-type strain, Δ*SsCut1-3*, Δ*SsCut1-3-C8* and Δ*SsCut1-3-C12* strains on PDA medium at 20 °C in complete darkness. Photographs were taken at 36 h post-inoculation (hpi). (B) Sclerotial primordium of wild-type strain, Δ*SsCut1-3*, Δ*SsCut1-3-C8* and Δ*SsCut1-3-C12* strains on PDA medium at 20 °C in complete darkness. Photographs were captured at 3 days post-inoculation (dpi). (C) Sclerotial formation of wild-type strain Δ*SsCut1-3*, Δ*SsCut1-3-C8* and Δ*SsCut1-3-C12* strains on PDA medium at 20 °C in complete darkness. Photographs were taken at 7 days post-inoculation (dpi). (D) Growth rate of all the strains growing on a PDA plate at 20 °C in complete darkness. (E) Comparation of sclerotia weight of strains growing on a PDA plate at 20 °C for 7 days in complete darkness. (F) Sclerotia number per plate of strains growing on a PDA plate at 20 °C for 7 days in complete darkness. Three independent replications were performed for each treatment. Bars indicate ± SE. Statistical differentiation was evaluated by t-test. Different letters on a graph indicate significant differences, *p* < 0.01. Figure S4: All the knock-out mutants of *SsCut1* showing reduced virulence on detached oilseed rape leaves. (A) Lesions formation on oilseed rapeseed leaves inoculated with wild-type strain and *SsCut1* transformants, the photographs were taken at 24, 36, 48 h post-inoculation (hpi), respectively. (B) Statistical results of lesion area on oilseed rape leaves. Bars indicate ± SE. Statistical significance is indicated in the graph (one-way ANOVA): * *p* < 0.05, ** *p* < 0.01.
