# Supplementary material for: Sclerotinia sclerotiorum SsCut1 Modulates Virulence and Cutinase Activity
Source: J Fungi (Basel). 2022 May 20;8(5):526. doi: 10.3390/jof8050526 (PMC9143608; doi:10.3390/jof8050526)

Journal of *Fungi*

Supplementary Information: *Sclerotinia sclerotiorum SsCut1* modulates virulence and cutinase activity

Yingdi Gong[1,2], Yanping Fu[2], Jiatao Xie[1,2], Bo Li[1,2], Tao Chen[1,2], Yang Lin[2], Weidong Chen[3], Daohong Jiang[1,2], Jiasen Cheng[1,2*]

[1] State Key Laboratory of Agricultural Microbiology, Huazhong Agricultural University, China, Wuhan 430070, China
[2] The Provincial Key Lab of Plant Pathology of Hubei Province, College of Plant Science and Technology, Huazhong Agricultural University, Wuhan 430070, China
[3] United States Department of Agriculture, Agricultural Research Service, Washington State University, Pullman, WA 99164, USA
* Correspondence: jiasencheng@mail.hzau.edu.cn; Current Address: State Key Laboratory of Agricultural Microbiology, Huazhong Agricultural University, Wuhan 430070, China;Tel: +86 27 87280487

Figure S1: Exon and intron structure display of cutinase family genes of *S. sclerotiorum*. Exons and introns are indicated by red boxes and lines, respectively. The names of the *S. sclerotiorum* cutinase genes and intron-exon structures are indicated at the left and right sides, respectively. The number in the figures indicates the length of each intron and exon.

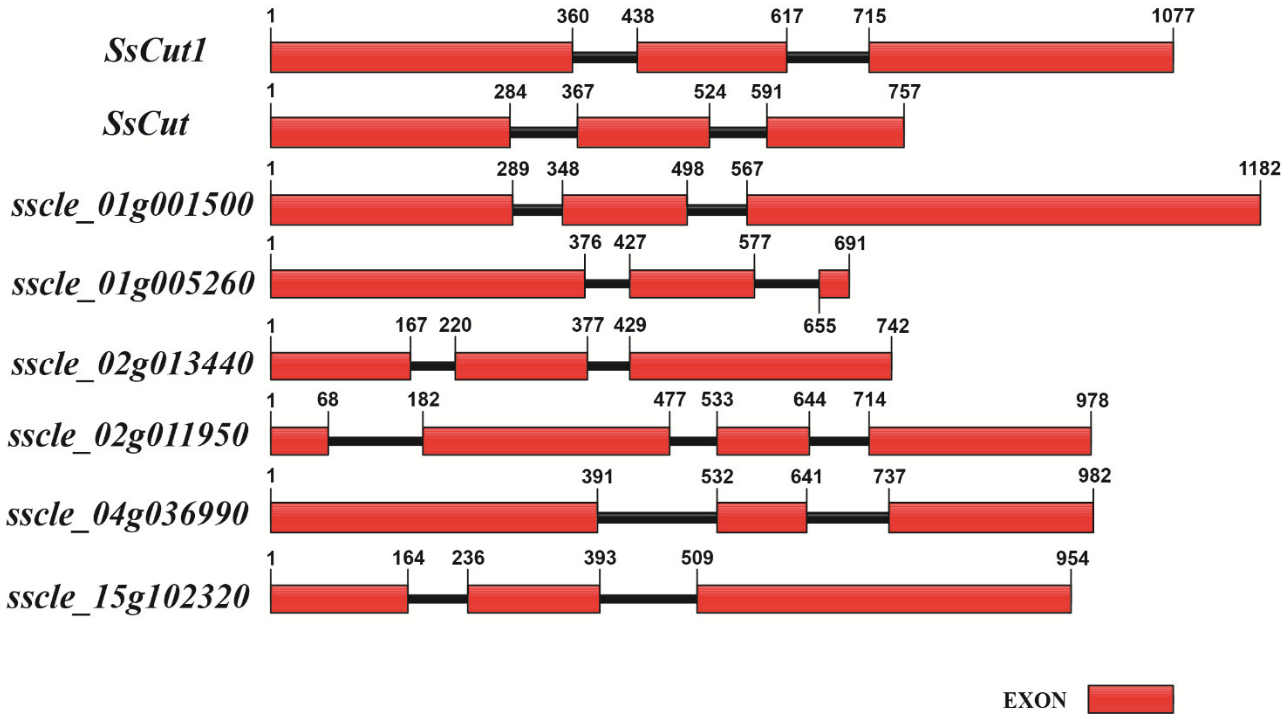

Figure S2: Analysis of *SsCut1* mutants. (A) Schematic diagram of *SsCut1* gene knock-out strategy. The different colored parts show the *HptII* cassette (white color parts), *SsCut1* (black box parts) and flanked sequences (grey box parts). (B) PCR validation of the *SsCut1* knock-out strains. The upstream of *SsCut1* overlapped Ptrp (Lanes 1, 5, 9, 13); the downstream of *SsCut1* overlapped trpC (Lanes 2, 6, 10, 14); Partial *HptII* fragment (Lanes 3, 7, 11, 15); the full-length *SsCut1* (Lanes 4, 8, 12, 16). Lane M, DL5000 marker (Takara, Dalian, China). (C) Southern blot analysis of the SpeI-digested genomic DNAs from wild-type strain, Δ*SsCut1-1*, Δ*SsCut1-3* and Δ*SsCut1-7* strains. Probe was labelled with alkaline phosphatase. Lane M, DNA marker λ-HindIII DNA Ladder (Takara, Dalian, China). (D) PCR validation of the *SsCut1* complementary strains. The full-length *SsCut1* (Lanes 1, 4, 7, 10); Partial *HptII* fragment (Lanes 2, 5, 8, 11); Partial G418 fragment (Lanes 3, 6, 9, 12). Lane M, DL2000 marker (Takara, Dalian, China).

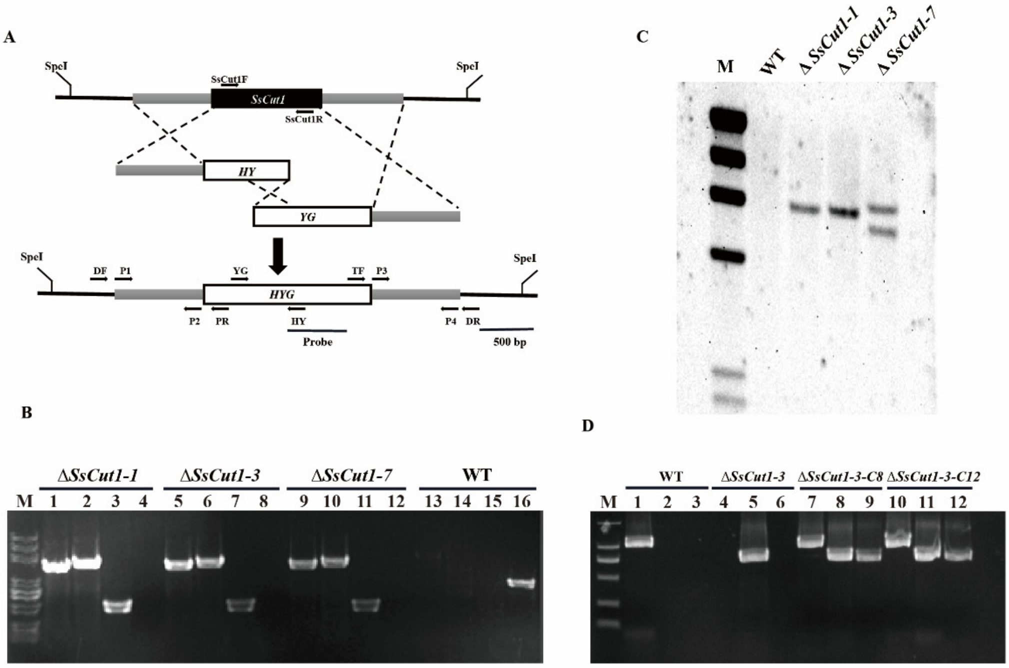

Figure S3: Colony morphology of WT, Δ*SsCut1-3*, Δ*SsCut1-3-C8* and Δ*SsCut1-3-C12* strains. (A) Growth of wild-type strain, Δ*SsCut1-3*, Δ*SsCut1-3-C8* and Δ*SsCut1-3-C12* strains on PDA medium at 20°C in complete darkness. Photographs were taken at 36 h post-inoculation (hpi). (B) Sclerotial primordium of wild-type strain, Δ*SsCut1-3*, Δ*SsCut1-3-C8* and Δ*SsCut1-3-C12* strains on PDA medium at 20°C in complete darkness. Photographs were captured at 3 days post-inoculation (dpi). (C) Sclerotial formation of wild-type strain Δ*SsCut1-3*, Δ*SsCut1-3-C8* and Δ*SsCut1-3-C12* strains on PDA medium at 20°C in complete darkness. Photographs were taken at 7 days post-inoculation (dpi). (D) Growth rate of all the strains growing on a PDA plate at 20°C in complete darkness. (E) Comparation of sclerotia weight of strains growing on a PDA plate at 20°C for 7 days in complete darkness. (F) Sclerotia number per plate of strains growing on a PDA plate at 20°C for 7 days in complete darkness. Three independent replications were performed for each treatment. Bars indicate ± SE. Statistical differentiation was evaluated by t-test. Different letters on a graph indicate significant differences, $P < 0.01$.

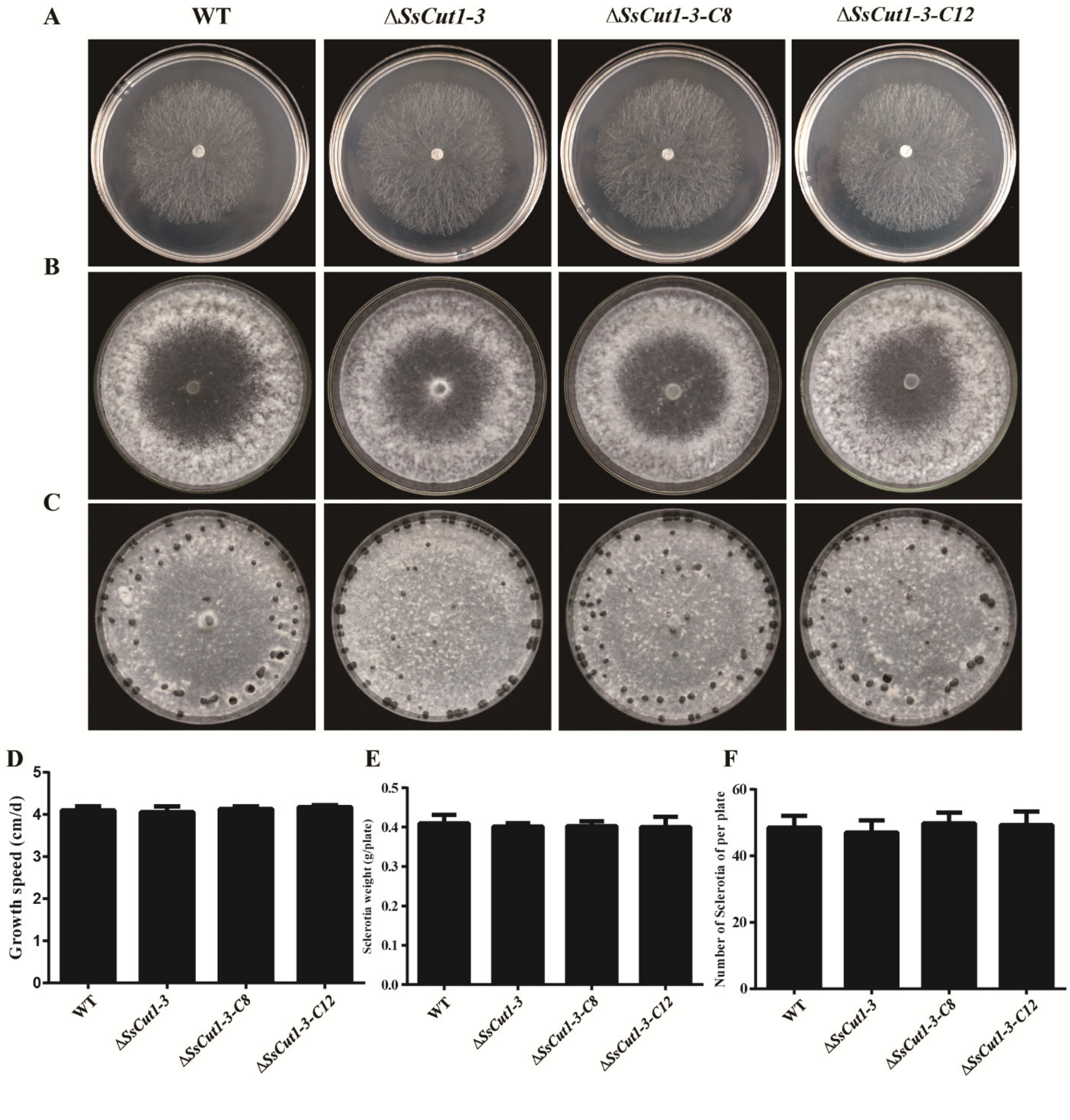

Figure S4: All the knock-out mutants of *SsCut1* showing reduced virulence on detached oilseed rape leafs. (A) Lesions formation on oilseed rapeseed leafs inoculated w Cutinase activity levels were examined with the enzyme-linked immunosorbent assay (ELISA) method ith wild-type strain and *SsCut1* transformants, the photographs were taken at 24, 36, 48 hours post inoculation (hpi), respectively. (B) Statistical results of lesion area on oilseed rape leaves. Bars indicate ± SE. Statistical significance is indicated in the graph (one-way ANOVA): * P < 0.05, ** P < 0.01.

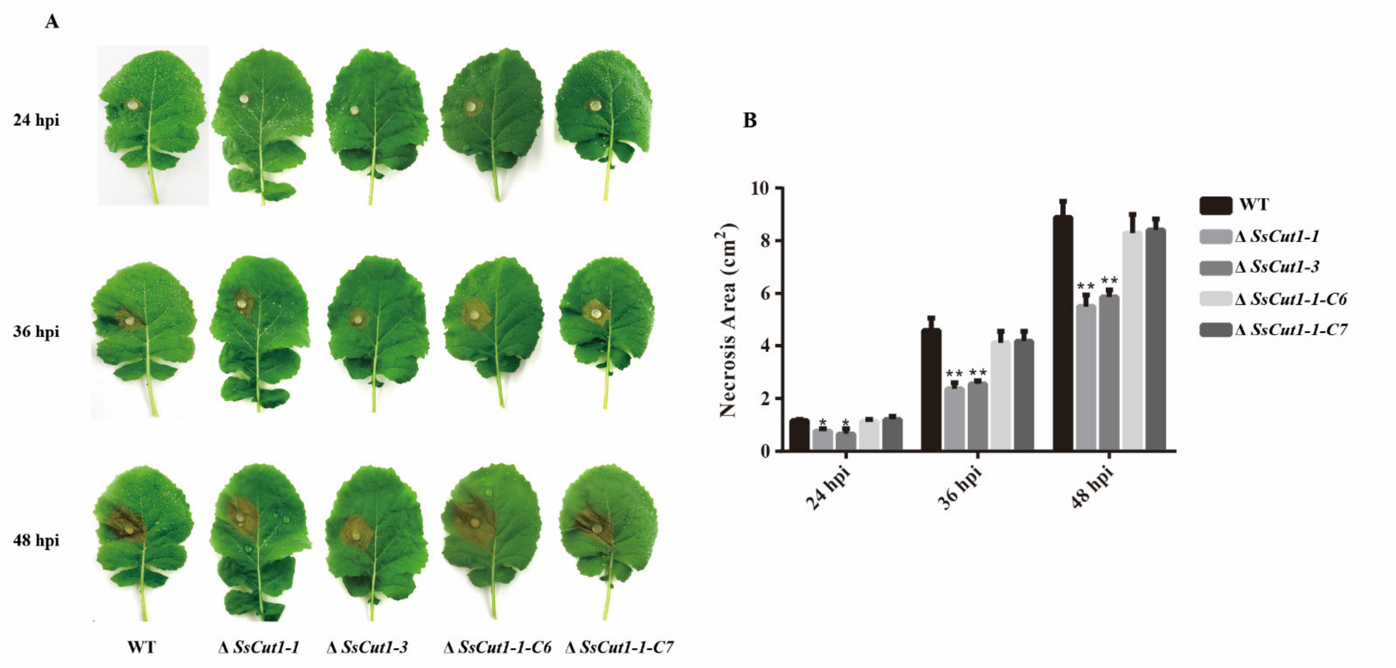

Supplement: Supplementary file 1 [file jof-08-00526-s001.zip › jof-1715385-supplementary.pdf]
